# Targeting LOX-1 Inhibits Colorectal Cancer Metastasis in an Animal Model

**DOI:** 10.3389/fonc.2019.00927

**Published:** 2019-09-19

**Authors:** Michela Murdocca, Rosamaria Capuano, Sabina Pucci, Rosella Cicconi, Chiara Polidoro, Alexandro Catini, Eugenio Martinelli, Roberto Paolesse, Augusto Orlandi, Ruggiero Mango, Giuseppe Novelli, Corrado Di Natale, Federica Sangiuolo

**Affiliations:** ^1^Department of Biomedicine and Prevention, Tor Vergata University, Rome, Italy; ^2^Department of Electronic Engineering, Tor Vergata University, Rome, Italy; ^3^Centro Servizi Interdipartimentale STA, Tor Vergata University, Rome, Italy; ^4^Department of Chemical Science and Technology, Tor Vergata University, Rome, Italy; ^5^Cardiology Unit, Department of Emergency and Critical Care, Policlinic of Tor Vergata, Rome, Italy

**Keywords:** colorectal cancer, LOX-1, shRNAs, xenograft model, neo-angiogenesis, metastatic cancer, VOCs analysis, gas sensor array

## Abstract

Recurrence and metastasis are the primary causes of mortality in patients with colorectal cancer (CRC), and therefore effective tools to reduce morbidity and mortality of CRC patients are necessary. LOX-1, the ox-LDL receptor, is strongly involved in inflammation, obesity, and atherosclerosis, and several studies have assessed its role in the carcinogenesis process linking ROS, metabolic disorders and cancer. We have already demonstrated *in vitro* that LOX-1 expression correlates to the aggressiveness of human colon cancer and its downregulation weakens the tumoral phenotype, indicating its potential function as a biomarker and a target in CRC therapy. Here we further investigate *in vivo* the role of LOX-1 in colon tumorigenesis by xenografting procedures, injecting nude mice both subcutaneously and intravenously with human high grade metastatic colorectal cancer cells, DLD-1, in which LOX-1 expression has been downregulated by shRNA (LOX-1_RNAi_ cells). Histopathological and immunohistochemical evaluations have been performed on xenograft tumors. The experiments have been complemented by the analysis of the volatile compounds (VOCs) collected from the cages of injected mice and analyzed by gas-chromatography and gas sensors. After intravenous injection of LOX-1_RNAi_ cells, we found that LOX-1 silencing influences both the engraftment of the tumor and the metastasis development, acting by angiogenesis. For the first time, we have observed that LOX-1 inhibition significantly prevents metastasis formation in injected mice and, at the same time, induces a downregulation of VEGF-A165, HIF-1α, and β-catenin whose expression is involved in cell migration and metastasis, and a variation of histone H4 acetylation pattern suggesting also a role of LOX-1 in regulating gene transcription. The analysis of the volatile compounds (VOCs) collected from the cages of injected mice has evidenced a specific profile in those xenograft mice in which metastasis originates. These findings underline the role of LOX-1 as a potential target for inhibition of tumor progression and metastasis, enhancing current therapeutic strategies against colorectal cancer.

## Introduction

Colorectal cancer (CRC) is one of the most prevalent and widely studied cancers in the world, conferring significant morbidity, mortality, and cost to the public health system (1). Metastatic disease, most prominently to the liver and lung, develops in 30–40% of CRC patients. Thus, a better knowledge of the molecular basis of its onset and progression is needed to define preventive, diagnostic, and prognostic protocols.

LOX-1 has been first identified as a major receptor of oxidized-LDL (ox-LDL) and it is mainly expressed in endothelial cells and vascular-rich organs, playing a crucial role in cardiovascular diseases (2). Its altered expression is also associated with inflammation, high levels of cholesterol, atherosclerosis, diabetes, and obesity, which are components of the “metabolic syndrome” (3, 4).

Most relevantly, a positive correlation has been reported among LOX-1 expression and the carcinogenesis process (3–5). Specifically, previous studies have highlighted a positive link between increased level of ROS, lipid peroxides and carcinogenesis; in this context ox-LDL binding to LOX-1 increases ROS formation, contributing to oxidative DNA damage (5–8). Also, the meta-analysis of gene expression profiles of about 950 cancer cell lines, stored in the Gene Expression Atlas at the EMBL-EBI database (http://www.ebi.ac.uk/gxa/gene/ENSG00000173391#), reveals that LOX-1 is upregulated in 57% of bladder and cervix cancer cells, 11% of mammary gland cancer cells, 10% of lung cancer cells and, importantly, in 20% of CRC cells (3). Significantly, other studies have shown that a high LOX-1 expression represents a substantial prognostic factor in various type of cancers, such as advanced stage prostate cancer, squamous non-small cell lung cancer, and gastric and pancreatic cancer (9–12). The depletion of LOX-1 receptors protects against tumorigenicity, tumor motility, and growth (3).

Epidemiological studies reveal that those individuals showing high levels of circulating ox-LDL, in addition to atherosclerotic plaques, are more susceptible to CRC (5, 7). This hypothesis is further supported by the observation that the inhibition of cholesterol production by statins administration protects against cancerogenesis, with a 47% relative reduction in the risk of CRC (13, 14). Thus, LOX-1 potentially serves as a robust linkage among ROS, metabolic disorders, and cancer (15).

The role played by LOX-1 in tumorigenesis also takes place through angiogenesis, which represents an early to mid-stage event in many human cancers. In particular LOX-1 upregulation increases VEGF-A165 expression (8, 16).

In addition, *in vitro* studies demonstrated that ox-LDLs promote pathways related to β-catenin (17), a protein involved in colon cancer insurgence that in turn is able to enhance HIF-1α mediated transcription, thereby promoting cell survival and adaptation to hypoxia (18).

The known relationship between cancer and volatile compounds (VOCs) has motivated the study of tumor progression through the changes of VOC profile. The volatile portion of metabolism products, sometimes called volatilome, is supposed to reflect the metabolic changes due to a plethora of diverse factors, such as inflammation, necrosis, cancer degeneration, or alteration of microbiota. The relationship between volatilome composition and tumors has been observed in many experiments, both *in vitro* and *in vivo* (19–22).

In the last few years, VOC profile analysis for colorectal cancer diagnosis has been investigated, leading to promising results (23–27). These kind of studies are still mostly empirical; however, the analysis of VOCs provides an independent and simple method to evaluate the rate of metabolic processes in living beings, so it is very useful to monitor the evolution of tumors.

Recently, our research has shed light on the role of the LOX-1 receptor as a novel biomarker and molecular target to improve current therapeutic strategies of CRC (28). In particular, we have shown that LOX-1 expression correlates to the aggressiveness of human colon cancer and that a downregulation of its expression *in vitro*, especially in a metastatic colon cell line (DLD-1), weakens the tumoral phenotype in terms of cell growth and motility. Also, *in vitro* LOX-1 modulation influences the presence of peculiar volatile organic compounds (VOCs) (29). Moreover, the variation of LOX-1 expression elicits the variation of histone H4 acetylation, thus suggesting a role of LOX-1 in regulating gene transcription in colon cancer (29).

In this paper, we explore *in vivo* the role of LOX-1 in colon tumorigenesis using two different xenografting procedures: subcutaneous and endovenous. Our results show that LOX-1 is involved in tumor engraftment and metastasis development, also by inducing the neo-angiogenic process. Moreover, analyzing the volatile compounds obtained from the cage of injected mice, we have studied the changes in volatilome composition by Gas Chromatography Mass Spectroscopy (GC/MS) (30) and Gas sensor array (28). The obtained results show that the modulation of LOX-1 expression correlates to VOCs profile *in vivo*.

## Methodology

### Subcutaneous Xenograft and Experimental Metastasis Mouse Models

3.5 × 10^6^ of DLD-1 cells (ATCC: CCL-221TM) in which LOX-1 has been knocked down by RNAi (LOX-1_RNAi_) and a scramble (scramble_RNAi_), diluted in 150 μl of PBS, have been transplanted subcutaneously into the right flank of CD-1 male nude mice aged 5 weeks (*n* = 10 per group). Regarding the metastasis mouse model, 4 × 10^6^ cells have been injected intravenously via tail vein of male nude mice aged 5 weeks (*n* = 8 per group). Saline solution injected mice have been evaluated both for subcutaneous (*n* = 10 per group) and intravenous (*n* = 10 per group) procedures. Mice tumor volume was measured by caliper every week for 28 days from the mass insurgence until mice sacrifice. Also, the body weight of the mice was monitored and tumor masses were measured. Further information is in Supplementary Materials and Methods.

All animals injected subcutaneously and intravenously were sacrificed after 4 and 11 weeks, respectively, and tumor masses weighted. Further information is in Supplementary Materials and Methods. All mice tumor masses, lymph nodes, and major organs were preserved in 4% buffered formalin, embedded in paraffin, sectioned and stained with H&E.

### Tumor Morphometric Analysis

Morphometric evaluation of total tumoral area (mm^2^), necrosis area (mm^2^), percentage of necrosis, and residual tumoral area (mm^2^) has been performed on images captured by ACT-1 software (Nikon, Japan) connected to a Nikon microscope (EclipseE600), and analyzed by Scion Image software (Scion Corporation, Frederick, MA). Blinded evaluation of capillary density (vessels per mm^2^) at 40× magnification was performed on 10 independent fields/animals by three observers in 20 serial sections, with intervariability <5%. Necrotic and hemorrhagic areas have been excluded from the vessel count.

### Metastasis Evaluation

At autopsy, the left and right inguinal lymph nodes, axillary lymph nodes, liver, lung, and spleen were removed. All the serial sections (20 sections/organ) of 5 μm stained with H&E have been used for histopathological evaluation of the presence of metastasis and micro-metastasis and evaluated histologically by light microscopy. Any number of tumor cells present in the lymph node or in organ was considered as evidence of metastatic spread.

### Immunohistochemistry (IHC)

Serial four micron sections of xenograft tumors have been immunostained for Ki67 (MoAb, Dako, Denmark), VEGF-A165 (A-20, Santa Cruz biotechnology, U.S.), Acetyl Histone H4 (Upstate, NY), α-SMA (Dako, Denmark), CD31 (ab124432, Abcam), β-catenin (sc-7963, Santa Cruz Biotechnology, Inc.), and HIF-1α (MA1-16504, Affinity Bioreagents) following the streptavidin-biotin method, as previously described (29). Slides were independently examined by three pathologists. All cases were digitally scanned by iScan (BioImagene, Now Roche-Ventana) with Scanning Resolution 0.46 μm/pixel at 20× or 40×. Tissue staining was semi-quantitatively graded for intensity as negative/weak: 0, moderate: 1 and strong: >2. The cell positivity was also scored as <10% (0), from 10 to 25% (1) from 26 to more than 50% (2). The final score was calculated by adding both partial scores. For the evaluation of results, the Chi square test was performed. The density of vessels, immunostained by CD31 and α-SMA, was determined by calculating the tube number per 40× field of view. Five areas were chosen and an arithmetic mean was obtained. The microvessel density quantification was performed manually, by three independent experts in the field. The image processing was performed with Lucia G System and the statistical analysis was performed with SPSS Software ver. 20.

### Volatile Compounds Sampling

Mice were kept for 30 min without food and then moved in the experiment room and placed in a polypropylene box (KIS T box XXS- ABM Italia S.p.A.). The box was large enough to allow for normal physical activity. The lid of the box has been endowed with an inlet suitable for the insertion of either the Solid Phase MicroExtraction (SPME) sampler or the gas sensor array sampling tube. The total air released by the mice inside the measurement cage was collected on a SPME and then analyzed with the Gas Chromatography Mass Spectroscopy (GC/MS). The SPME fiber was a 50/30 μmDivinylbenzene/Carboxen/PDMS (SUPELCO, Bellefonte, PA, USA). In all measurements, the fiber was kept in the sampling box for 1 h, and analyzed over 3 h after their collection.

To standardize the headspace formation, the VOCs collection always began 15 min after the mouse entered the cage. The temperature and humidity of the experiment room was kept constant during the whole experiment.

The box floor was coated, before each measurement, with filter paper sheets (Biosigma srl) to collect solid and liquid dejection.

### Gas Chromatography Mass Spectroscopy (GC-MS)

The GC/MS is a Shimadzu GCMS-QP2010 (Kyoto, Japan) equipped with a capillary column EQUITY-5 poly (5% diphenyl/95% dimethyl siloxane) (SUPELCO, Bellefonte, PA, USA). The size of the column is 30 m length × 0.25 mm I.D. × 0.25 μm thickness. The VOCs were desorbed from the SPME in splitless injection mode at 250°C for 3 min in the GC injection port. Compounds have been putatively identified using both NIST 127 and NIST 147 libraries. The identity of the compounds found in more than 80% of samples were confirmed comparing the mass spectra with those of pure standard obtained from Sigma Aldrich (31). Pure compounds were used as received without any further purification. Additional method details can be found in Supplementary Materials and Methods.

### Gas Sensor Array

The gas sensor array was an ensemble of twelve quartz microbalances (QMB). These sensors detected the mass change (Δm) in absorbing layer deposited onto the surface of the quartz. The sensor signal is the change of the frequency (Δf) of the electric output signal of an oscillator circuit driven by the quartz. In the regime of small perturbations, Δm and Δf are linearly proportional. The adopted QMBs have a fundamental frequency of 20 MHz, corresponding to a mass resolution of the order of a few nanograms. The sensing materials are solid-state layers of porphyrinoids (porphyrins and corroles) (32). The baseline of sensor signals were measured in a constant flow of reference air made filtering ambient air with a CaCl_2_ trap. The difference of the sensor signals taken in reference air respect to sample air were used as the sensor response. The cage air was sampled for 3 min at the constant flow of 75 sccm.

## Results

### The Inhibition of LOX-1 Induce Necrotic Area Formation in Xenograft Tumors

In a previous study, after quantifying LOX-1 expression among several CRC cell lines, we focused our attention on two cell lines overexpressing LOX-1, HCT8 (non-metastatic), and DLD-1 (metastatic), in which we stably down modulated LOX-1 expression by RNAi (29).

Here in this study we decided to use DLD-1 cells, a colon cancer metastatic cell line, stably knocked down for LOX-1 (LOX-1_RNAi_ DLD-1) as a model of colon cancer insurgence and spreading in nude mice. Before starting, the downregulation of LOX-1 mRNA expression was again confirmed by RT-qPCR (Supplementary Figure 1).

Mice have been subcutaneously injected with LOX-1_RNAi_ DLD-1 and scramble_RNAi_ DLD-1 cells in the right flank, as described in Materials and Methods. In parallel, a group of animals were injected with a saline solution. Tumor volume was monitored once a week for 4 weeks and measured by a caliper. Mice body weight was also measured once a week. For both, no significant differences were reported among the two groups of mice (Supplementary Figure 2). After 28 days, mice were sacrificed and, after measuring and weighting the xenograft tumoral mass, tumors, and organs were collected for histochemical and histopathological evaluations. No metastasis foci have been evidenced in liver, lung, kidney, as in lymph nodes.

Even if the tumor weight and volume did not differ significatively between two mice groups (Supplementary Figure 2), the histopathological evaluation of the tumoral mass showed significant differences. The tumoral area formed by LOX-1_RNAi_ was significantly reduced in respect to the tumoral area formed by scramble_RNAi_ cells (*P* = 0.002) (Table 1). In addition, the necrosis area (*% necrosis* = *necrosis/total tumoral area*) was significantly higher in tumors formed by LOX-1_RNAi_ cells. These results indicate that the weight of LOX-1 _RNAi_ xenograft, as compared to scramble_RNAi_ tumors, do not display significant differences because the necrosis has a weight and a volume comparable to scramble_RNAi_ tumor tissue area. Thus, in order to evaluate if the delayed growth of xenograft tumor in LOX-1_RNAi_ DLD-1 injected mice is connected to an impaired LOX-1-dependent neo-angiogenesis, we have evaluated the number of neo vessels in xenograft tumors and its surrounding tissues together with the expression of VEGF-A165, a well-known soluble factor involved in tumoral angiogenesis.

**Table 1:** Morphometric analysis of tumor area in LOX-1_RNAi_ and scramble_RNAi_ mice evaluated on H.E. sections of the xenograft tumors.

	**n° animals**	**Total tumoral area (mm^**2**^)**	**Necrosis (mm^**2**^)**	**% Necrosis**	**Residual tumoral area (mm^**2**^)**
**LOX-1**_**RNAi**_	10	58.89 ± 10.3	25.79 ± 4.6	42.58 ± 2.40	34.10 ± 6.1
**Scramble**_**RNAi**_	10	81.35 ± 17.24	27.06 ± 6.8	32.84 ± 2.35	54.29 ± 10.9

*All values are referred to VM ± SEM (necrosis area: LOX-1_RNAi_ DLD-1 vs. scramble_RNAi_; P = 0.002)*.

### New Blood Vessels Formation Is Decreased in LOX-1_RNAi_ DLD-1 Xenograft Tumors and Correlated to a Decrease of VEGF-A165 Expression

**Figure 1:**
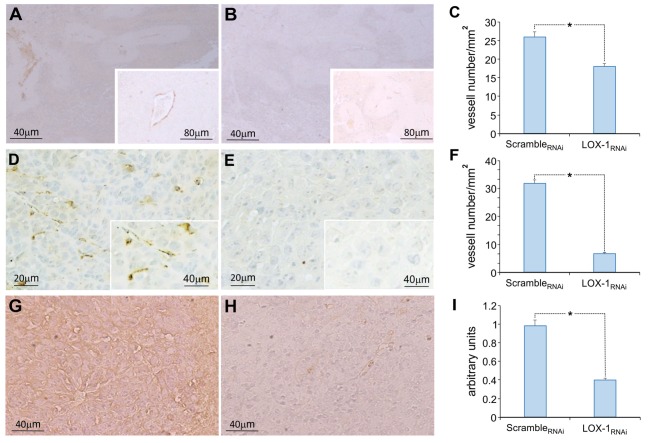
Representative images of the immunohistochemical analysis of α-SMA, CD31, and VEGF-A165 expression in xenograft tumoral tissues of mice injected subcutaneously. Neo vessels evidenced by α-SMA immunohistochemistry in scramble_RNAi_ **(A)** and in LOX-1_RNAi_ **(B)** xenograft tumors. Scale bar 40 μm. A representative neo vessel in inset (scale bar 80 μm). **(C)** Graph showing the number of vessels identified by α-SMA antibody in LOX-1_RNAi_, significantly reduced respect to those observed in scramble_RNAi_ xenograft tumors (*P < 0.01). Neo vessels evidenced by CD31 immunohistochemistry in scramble_RNAi_ **(D)** and in LOX-1_RNAi_ **(E)** xenograft tumors. Scale bar 20 μm. A representative neo vessel in inset (scale bar 40 μm). Graph showing the number of vessels identified by CD31 antibody in LOX-1_RNAi_, significantly reduced respect to those observed in scramble_RNAi_ xenograft tumors (*P < 0.01) **(F)**. Representative images of the immunohistochemical analysis of VEGF-A165: tumoral mass in scramble_RNAi_ **(G)** and in LOX-1_RNAi_ **(H)** xenograft tumors. Scale bar 40 μm. Graph representing the quantification of VEGF-A165 signal, significantly decreased in LOX-1_RNAi_ respect to scramble_RNAi_ tumors **(I)** (*P < 0.01).

Blood vessels maturation is a crucial factor for colorectal cancer growth. Therefore, with the aim of assessing the role played by LOX-1 in neo-angiogenesis, histopathological and immunohistochemical analyses were performed for α-SMA, CD31 and VEGF-A165 expression on the tumoral mass and surrounding tissues of mice subcutaneously injected. The number of vessels has been identified by evaluating α-SMA expression in intratumoral and peritumoral tissues of scramble_RNAi_ and LOX-1_RNAi_ xenograft tumors (Figures 1A,B).

High number of neo vessels were present in scramble_RNAi_ xenograft tumors, and the aspect was sometimes discontinuous, especially in small caliber vessels. On the contrary, a very few number of vessels with a small caliber were evidenced in LOX-1_RNAi_ xenograft tumors (Figure 1B). Specifically a significantly reduced number of vessels has been observed in LOX-1_RNAi_ tumors compared to scramble_RNAi_ ones (LOX-1_RNAi_ vs. scramble_RNAi_: *P* < 0.01) (Figure 1C). Also IHC staining for CD31 (Figures 1D,E) showed a discontinuous pattern confirming the presence of blood neo vessels in a higher number in scramble_RNAi_ tumors with respect to LOX-1_RNAi_ ones (*P* < 0.01) (Figure 1F), in accordance with data obtained by α-SMA staining.

Finally, we have analyzed the expression of VEGF-A165 by immunohistochemistry performed on the same xenograft tumor tissues. The immunostaining of VEGF-A165 was observed in the cytoplasm of tumor cells, as well as in endothelial and in some stromal cells in scramble_RNAi_ DLD-1 xenograft tissues (Figure 1G). On the contrary, the expression of VEGF-A165 resulted strongly diminished (2.5 times) in LOX-1_RNAi_ xenograft tumor tissues, in which LOX-1 has been downregulated (Figure 1H). The difference resulted to be statistically significant (*P* < 0.01) (Figure 1I).

### LOX-1 Silencing Influences β-Catenin and HIF-1α Expression

In order to understand the role of LOX-1 in promoting tumor growth and angiogenesis, we examined the expression of β-catenin and HIF-1α in the same tissues.

**Figure 2:**
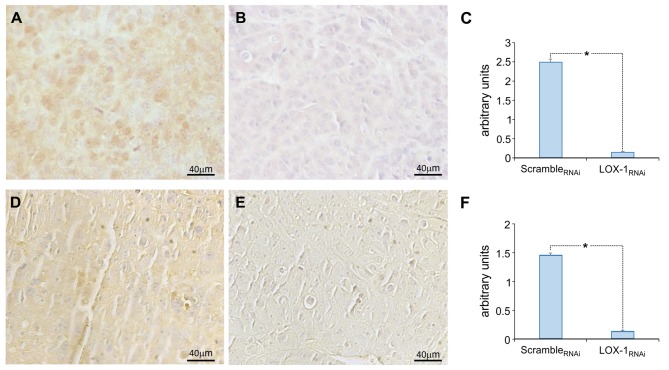
Representative images of the immunohistochemical analysis of β-catenin and HIF-1α expression. β-catenin expression in scramble_RNAi_ **(A)** and LOX-1_RNAi_ **(B)** xenograft tumors, and HIF-1α expression in scramble_RNAi_ **(D)** and LOX-1_RNAi_ **(E)** xenograft tumors. Scale bar 40 μm. Graphs show the significant decrease of β-catenin expression **(C)** and HIF-1α expression **(F)** in LOX-1_RNAi_ respect to scramble_RNAi_ tumors (*P < 0.01).

The immunohistochemical analysis of β-catenin is represented in Figure 2, showing a strong positive staining (3+) in the cytoplasm and 25% of positivity in the nucleus of scramble_RNAi_ tumor cells (Figure 2A). On the contrary, we have found an evident decreased expression of β-catenin in LOX-1_RNAi_ tumors (Figure 2B), confirming that LOX-1 positively regulates β-catenin expression. The difference was a statistically significant result (Figure 2C; *P* < 0.01**)**. Furthermore, the expression of HIF-1α was also found to be significantly decreased in LOX-1_RNAi_ tumors (Figures 2E,F) with respect to scramble_RNAi_ (Figure 2D), confirming published data on the role of β-catenin in HIF-1α transcription and expression (18).

### Silencing LOX-1 in DLD-1 Cells Inhibits Cell Proliferation and Induces a Modulation of Histone H4 Acetylation in Subcutaneously Induced Xenograft Tumors

In order to define the role of LOX-1 in proliferation and gene transcription modulation, we have performed IHC for Ki67 protein, a proliferative nuclear marker and histone H4 acetylation on xenograft tumor tissues.

**Figure 3:**
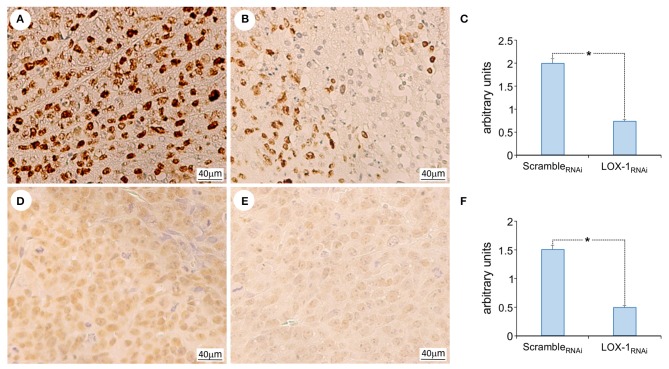
Representative images of the immunohistochemical analysis of Ki 67 and acetyl histone H4 expression. Ki 67 expression in scramble_RNAi_ **(A)** and LOX-1_RNAi_ **(B)** xenograft tumors, and acetyl histone H4 in scramble_RNAi_ **(D)** and LOX-1_RNAi_ **(E)** xenograft tumors. Scale bar 40 μm. Graphs show the significant decrease of Ki 67 expression **(C)** and acetyl histone H4 expression **(F)** in LOX-1_RNAi_ respect to scramble_RNAi_ tumors (*P < 0.01).

The immunohistochemistry analysis for Ki67 (Figures 3A,B) indicated that the intensity and the number of positive cells are significantly reduced in LOX-1_RNAi_ (Figure 3C), as compared to scramble_RNAi_ tumors. Moreover, the inhibition of LOX-1 induces a 2.5-fold decrease in LOX-1_RNAi_ tumor tissues of histone H4 acetylation pattern (Figures 3D–F) as compared to the scramble ones, suggesting a role of LOX-1 in histone H4 DNA acetylation and transcription.

### Volatile Compounds Are Quantitatively Different Among Mice Subcutaneously Injected With Saline and DLD-1 Cells

The total volatile compounds released by mice were collected and analyzed with the Gas chromatograph/Mass spectrometer (GC/MS).

In total, 79 different VOCs were found in the collected samples (Supplementary Table 1). Most of these VOCs appears only in few cases while 12 compounds have been found in more than 80% of all samples (Supplementary Table 2). All VOCs were identified by library database comparison, except the 12 recurrent compounds whose putative identity were confirmed by a comparison of the elution time and the mass spectra found in samples with those of standard compounds.

The comparison of the abundances of VOCs in the different samples was limited to the recurrent compounds; these are long chain aldehydes and methylated hydrocarbons. It is important to note that the selection of the recurrent VOCs was performed avoiding compounds found exclusively in one group of animals.

Supplementary Figure 3 shows the statistical distribution of the abundance of the selected VOCs in the three groups of mice (injected with scramble_RNAi_, LOX-1_RNAi_ and saline solution) measured at different days after the injection (1, 6, 13, and 20 days). The abundance of the VOCs is statistically undistinguishable between LOX-1_RNAi_ and scramble_RNAi_ DLD-1 injected mice, with the exception of the abundance of octane-4-methyl and decane between saline and DLD-1 injected mice (*P* < 0.001).

### LOX-1 Confers an Advantage in Metastasis Formation

**Figure 4:**
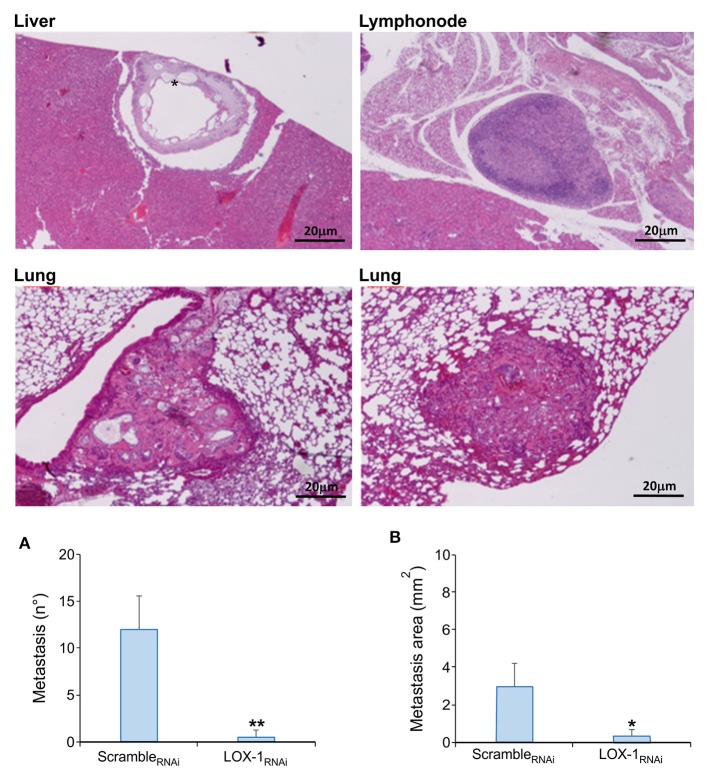
Representative images of murine organs evaluated on H&E sections. Metastasis foci have been evidenced in liver, lung and lymph nodes in mice endovenously injected with scramble_RNAi_ DLD-1 cells. Metastatic repetitions with mucinous aspect were found in all liver of scramble_RNAi_ mice (*). Scale bar 20 μm. Quantitative analysis of metastasis number **(A)** and metastatic area **(B)** in tissues obtained from mice endovenously injected with scramble_RNAi_ and LOX-1_RNAi_ cells. Metastasis number and area are significantly reduced in LOX-1_RNAi_ injected mice respect to those injected with scramble_RNAi_ cells. **P < 0.02,*P < 0.05.

For a second time, DLD-1 cells, as well as saline solution, was injected intravenously via the tail vein in a nude mice cohort (*n* = 8 per group). After 11 weeks, the mice were sacrificed and their major organs harvested for histopathological analyses. The number of metastasis and the metastatic area foci was measured in target organs, such as lung, liver, spleen and lymph nodes (Figures 4A,B).

Histopathological observations on tumor tissues evidenced the presence of multiple metastasis foci in lung and liver of all the mice injected with scramble_RNAi_ DLD-1 cells, and in three mice out of eight metastasis were evidenced also in lymph nodes (Table 2). On the contrary, the number of metastasis were significantly lower in mice injected with LOX-1_RNAi_ cells (**P < 0.02), as shown in Figure 4A. Only one mouse injected with LOX-1_RNAi_ cells presented metastatic foci in lung and in 2 lymph nodes, the other seven mice do not display any metastatic foci in the LOX-1_RNAi_-injected mouse. Moreover, the extension area of the metastatic repetitions was lower than in those injected with scramble_RNAi_ (0.21 ± 0.21 vs. 3 ± 0.72 mm^2;^*P < 0.05), as shown in Table 2 and Figure 4B. Interestingly, all scramble_RNAi_ metastatic foci in liver present aspects of mucinous cancers (Figure 4; see asterisk) the most aggressive colon cancer form, in which we evidenced mucin lakes comprising at least 50% of tumor metastasis areas with signet ring cells.

**Table 2:** Morphometric analysis of metastatic spread of LOX-1_RNAi_ and scramble_RNAi_ DLD-1 cells in murine organs evaluated on H&E sections.

**Treatment**	**Animal**	**Metastasis number**	**Metastasis VM ± SEM**	**Metastatic area (mm^**2**^)**	**Metastatic area VM ± SEM**	**Metastasis localization**
Scramble_RNAi_	MO13240	12		2.4		Lung, 2 lymph nodes
Scramble_RNAi_	MO13241	22		6.6		Lung, liver
Scramble_RNAi_	MO13242	5		1.3		Lung, liver
Scramble_RNAi_	MO13243	9	12 ± 3.2	1.8	3 ± 0.72	1 lymph node
Scramble_RNAi_	MO13244	8		1.5		Lung, liver
Scramble_RNAi_	MO13245	7		1.8		Lung, 1 lymph node
Scramble_RNAi_	MO13246	21		5.8		Liver
Scramble_RNAi_	MO13247	13		2.7		Lung, Liver
LOX-1_RNAi_	MO13248	0		0		
LOX-1_RNAi_	MO13249	0		0		
LOX-1_RNAi_	MO13250	0		0		
LOX-1_RNAi_	MO13251	0	0.37 ± 0.37	0	0.21 ± 0.21	
LOX-1_RNAi_	MO13252	3		1.7		Lung, 2 lymph nodes
LOX-1_RNAi_	MO13253	0		0		
LOX-1_RNAi_	MO13254	0		0		
LOX-1_RNAi_	MO13255	0		0		

### VOCs Pattern in Mice After Intravenously Cells Injection: Gas Chromatography and Gas Sensor Array

The total volatilome released by mice that underwent the intravenous injection was measured the day after the inoculation, and then after 4, 6, 9, and 11 weeks.

#### Gas Chromatography

In total, 67 VOCs were found in the mice cage air (Supplementary Table 3). These compounds were putatively identified from database comparison. The comparison of VOCs abundance were limited to the 11 compounds that were found in more than 80% of samples. The identity of these compounds was validated by a comparison of the elution time and the mass spectra found in samples with those of standard compounds (Supplementary Table 4). These compounds include aldehydes and alkanes, cyclic terpene, methylated alcohols, acids, and ketones. The chemical diversity is more rich than that found in subcutaneously injected mice.

Supplementary Figure 4 shows the statistical distribution of the abundances in the three groups of mice (injected with scramble_RNAi_, LOX-1_RNAi_ and saline solution) and at different weeks from the injection (4, 6, 9, and 11). After 4 weeks, mice injected with the scramble_RNAi_ DLD-1 showed an increase in the production of volatile metabolites (*P* < 0.001). Importantly, mice injected with saline and LOX-1_RNAi_ are not statistically different. This behavior is in agreement with the accelerated growth rate of the injected cells and their metastatic action.

Two of these compounds (octane-4-methyl and nonanal) are recurrent in both the experiments. These compounds are also released by saline injected mice. However, while in this group the abundances are uncorrelated (*r* = 0.48), a correlation evidently appears in LOX-1_RNAi_ and scramble_RNAi_ injected mice. Interestingly, the ratio of production of nonanal to octane-4-methyl depends on the injection method. In particular, the emission of octane-4-methyl to nonanal, increases in mice endovenously injected.

**Figure 5:**
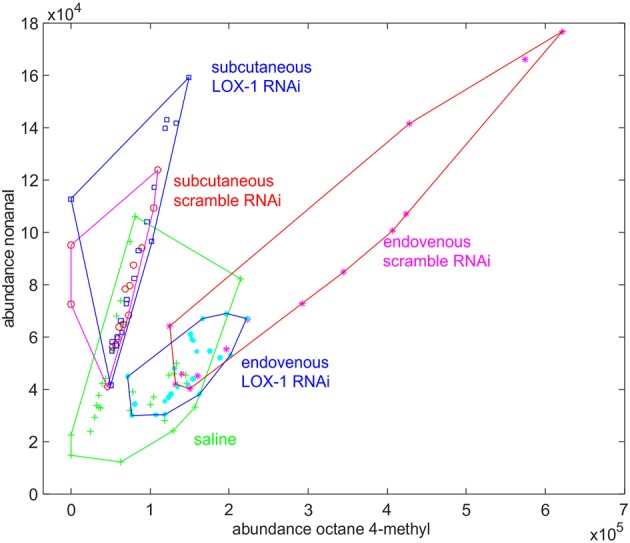
Plot of the abundance of nonanal vs. the abundance of octane-4-methyl found in mice volatilome. In each group the data collected along the whole experiments are plotted. While the abundance of nonanal is a specific of experimental condition, the abundance of octane-4-methyl is larger in mice endovenously injected with scramble_RNAi_ cells.

Furthermore, while the abundance of nonanal is independent of both the injection method and injected cells, octane-4-methyl is produced in excess by scramble_RNAi_ mice, endovenously injected (Figure 5).

#### Gas Sensor Array

**Figure 6:**
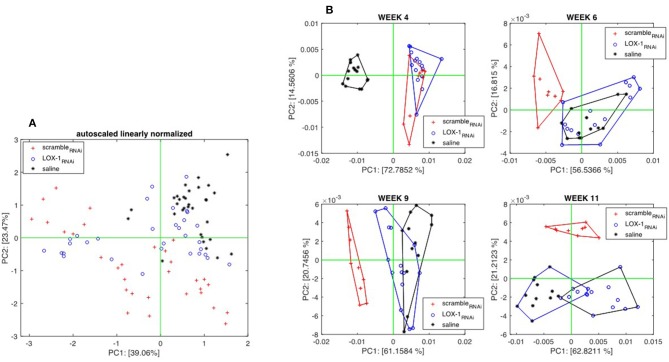
Principal component analysis of sensors data collected from endovenously injected mice. **(A)** Scores Plot of the first two principal components of the PCA calculated with the whole data set. Each group contains measures taken at different times. **(B)** Scores plots of PCA calculated with data collected in the individual experimental sessions held at weeks 4, 6, 9, and 11 after the injection. Score plots shows that, after the sixth week, the volatilome of the group of mice injected with scramble_RNAi_ cells are distinct from the others.

Gas Sensors were applied from the fourth week after the injection. Figure 6A shows the scores plot of the Principal Component Analysis (PCA) calculated with the whole sensor array dataset. Since the volatilome of mice were evolving, the data related to three groups are partially overlapped, and only a moderate separation between scramble_RNAi_ and LOX-1_RNAi_ can be appreciated. It is interesting to observe that the data of saline injected mice are more closely clustered than the other two groups. This behavior evidences that with DLD-1 cell injected mice, those only injected with saline show a reduced variability of the volatile compounds.

A better appraisal of the differences between groups is obtained when PCA is calculated with the data collected in the same measurement session. Figure 6B shows the score plots of individual session PCA models where a progression of the differences among the three groups can be observed. At week 4, LOX-1_RNAi_ and scramble_RNAi_ injected mice are separated from those injected with saline solution. At week 6, sensor array signals begin to distinguish between LOX-1_RNAi_ and *saline* mice groups from scramble_RNAi_. At weeks 9 and 11, *saline* and LOX-1_RNAi_ mice are progressively separated, but still kept well-distinct from scramble_RNAi_ mice.

The results of the sensor array are in good agreement with the GC/MS data, but with some differences. In particular, at week 4 sensors fail to capture the differences between scramble_RNAi_ and LOX-1_RNAi_ cell injected mice. This behavior suggests that the set of VOCs evidenced by GC/MS may not completely represent the volatile emissions from the mice. On the other hand, there might be other VOCs, which are not captured by the combination of the chosen SPME and GC/MS but are sensed by the gas sensors.

## Discussion

Invasion and metastasis are the deadly face of malignant tumors. Surgery represents the mainstay of treatment in early cases of CRC, but often patients are primarily diagnosed in an advanced stage of disease and sometimes also distant metastases are present. Five year survival rates are estimated to be between 85 and 90% for patients with localized cancer to colon or rectum. Survival decreases significantly for patients with distant metastasis, with 5 year survival of only 12.5%. Recurrence and metastasis are in fact the primary cause of mortality in patients with CRC. Neoadjuvant therapy is therefore needed. Recent therapeutic approaches that add epidermal growth factor receptor (EGFR) and VEGF targeted agents to standard chemotherapy have produced a prolonged overall survival (OS) of up to 30 months in patients with metastatic disease.

Within this context, a better understanding of the molecular mechanisms and biomarkers involved in early onset and in the metastatic process of CRC could play an important role to further improve the outcome of advanced CRC stage and metastatic CRC patients.

LOX-1, a marker for atherosclerosis (2), results to be overexpressed in several types of cancer, suggesting its role at the interface of atherosclerosis, metabolic disease and cancer (3, 4, 6–8, 33).

In particular, LOX-1 is overexpressed in the presence of inflammatory stimulus by PCSK9, an enzyme involved in cholesterol homeostasis. In fact, in hypercholesterolemic states it is possible to reduce atherogenesis by silencing LOX-1 via PCSK inhibitor (34). In addition, some drugs commonly used for the treatment of type 2 diabetic patients, such as statins or antidiabetic agents, are able in the same time to promote the inhibition of endothelial LOX-1 expression (35). Further study has demonstrated in *knockout* mice that the abrogation of LOX-1 causes a strong inhibition of rate-limiting enzymes involved in lipogenesis, including fatty acid synthase, ATP-citrate lyase, acetyl-coenzyme A carboxylase alpha, stearoyl-CoA desaturase 1, and Elongation of very long chain fatty acids protein family member 6, thus suggesting that LOX-1 may have a novel function as a potent regulator of lipogenesis (6).

Also, our previous studies on human tissues have evidenced a strong up-regulation of LOX-1 in human colon cancer tissues (29).

In the present work we evaluate the contribution of LOX-1 in tumor development, in metastasis formation and organ colonization *in vivo* by using a human high grade metastatic colon carcinoma (Duke's C) cells in which LOX-1 has been down modulated. For this purpose, we firstly injected LOX-1 downregulated DLD-1 cells (LOX-1_RNAi_) subcutaneously in a cohort of nude mice. While mice body weight, mean tumor weight and volume don't exhibit significant differences among the two cohorts of mice, the histopathological evaluation of the tumoral mass shows that the tumoral areas are significantly less extended in LOX-1_RNAi_ compared to scramble_RNAi_ xenograft tumors. More importantly, the necrosis area in LOX-1_RNAi_ tumors is significantly higher than scramble_RNAi_ ones, indicating that angiogenesis is not supporting tumor growth. This constitutes the clinically-relevant mechanism acted by LOX-1, suggesting that targeting neoangiogenesis through LOX-1 inhibition could represent a new strategy to control tumor growth and invasion. These results underlie the fact that the tumor weight does not differ, because the necrosis has a weight and a volume comparable to the tumor tissue, and therefore the weight and the dimension do not show any significant differences. Moreover, these data also match with a reduced expression of Ki67, VEGF-A165, and HIF-1α markers in LOX-1_RNAi_ mice tumors. The importance of tumor angiogenesis in the growth, progression, and metastasis of solid tumors in widely known. VEGF-A165 in particular is one of the most critical proteins to influence the angiotumoral dynamics with cell signaling response in proliferation and metastasis (36). Published data obtained with the antineoplastic treatment (bevacizumab) on a mouse xenograft model demonstrated a tight link between VEGF, neovessel formation, α-SMA and CD31 expression (37). In our model, these data are supported by the downregulation of VEGF-A165 and HIF-1α expression observed in LOX-1_RNAi_ tumor, demonstrating a direct link among LOX-1, VEGF-A165, and neo-angiogenic process involved in tumor formation and progression. Also, α-SMA, and CD31 expression evidences a significantly reduced number of vessels and pericytes in LOX-1_RNAi_ xenograft tumor compared to scramble_RNAi_ ones. In addition, LOX-1 influence the proliferative rate of neoplastic cells, as observed by immunohistochemistry analyses of Ki67, a marker of proliferation and tumor behavior, confirming the role of this receptor in the regulation of the proliferative rate of neoplastic cells.

Here we also report evidence of the pro oncogenic role of LOX-1 in supporting tumor growth, regulating the β-catenin expression. β-catenin is the most important regulator of proliferation, cell-cell adhesion, migration and epithelial-mesenchymal transition (EMT). The accumulation of β-catenin and the alteration of its degradation leads to colon cancer insurgence (38). Our results reporting a down modulation of the expression of β-catenin in LOX-1_RNAi_ xenograft tumors point out the importance of LOX-1 in colon cancer insurgence and progression. Furthermore, these results suggest that the β-catenin levels could be modulated by LOX-1, interfering with the function of the tumor suppressor APC, which in colon cancer is usually present in a truncated form. In DLD-1 cells, APC is deleted, but this mutation does not alter β-catenin degradation (39). Thus, the overexpression of β-catenin that we observe in scramble_RNAi_ tumors is not related to an accumulation of its products in the cytoplasm, but it could account to a high LOX-1 expression.

Furthermore, concomitantly to a reduced proliferative rate, knocking down LOX-1, we have also detected a modulation of histone H4 acetylation in LOX-1_RNAi_ tumor tissues, suggesting that a metabolic factor such as LOX-1 could modulate DNA acetylation and thus drive the gene expression pattern in tumors. In fact, histone H4 acetylation is one of the major regulation mechanisms of DNA transcription and determines chromatin accessibility with effects on gene expression. Some of the anticancer therapies act as HDAC inhibitors, the enzyme responsible for DNA deacetylation; this highlights the fundamental role of acetylation in tumor progression.

The impact of these findings is further strengthened by the observations that we made in a cohort of nude mice in which LOX-1_RNAi_ cells were intravenously injected. In these mice, we observed a significant inhibition of metastasis formation. Only one of these mice showed one metastasis in lung and two in lymph nodes. The small number of mice does not allow for a reliable statistical generalization of the results, however, the striking difference in metastasis development is in favor of the hypothesis of the role of LOX-1 in tumor progression. Importantly, all mice injected with scramble_RNAi_ cells showed metastasis in liver with aspects of mucinous cancers, the most aggressive colon cancer form. In fact, patients with mucinous colon cancer often have a poor prognosis and these tumors are prone to form metastasis, because mucin-associated carbohydrate plays a role in adhesive inter-structures that facilitate the survival of the metastatic clone (40).

The analysis of volatile compounds provides further evidence of the influence of LOX-1 in the progression of the tumor. Subcutaneously and endovenously injected mice have demonstrated to evolve differently, and this difference associated with the injection protocol has been found in the VOCs profile. The analysis was confined to the most recursive VOCs found in at least 80% of samples. In this way, episodical phenomena and random variations have been ruled out.

GC-MS analysis shows that mice injected with scramble_RNAi_ cells are characterized by the largest emission of volatile compounds. This behavior is particularly evident in endovenously injected mice while only a limited correlation has been found in mice treated with subcutaneous injection. For both the injection methods, all animals show the same kind of VOCs. Thus, the injection of DLD1 cells do not produce novel additional VOCs but a modulation of the abundance of the volatile metabolites that are usually emitted by mice.

The chemistry of the VOCs in the two experiments (i.e., subcutaneous and endovenous injection) is different and only two VOCs are common to both the experiments: octane-4-methyl and nonanal. The association of these compounds with cancer was found in previous studies. Octane-4-methyl was found at altered concentrations in the breath of patients affected by colorectal cancer (41) and lung cancer (42), while the content of nonanal in breath has been related to lung cancer (30) and ovarian cancer (43). Furthermore, both these compounds are also present among those VOCs signaling the differentiation of pluripotent stem cells (44). Here, we found that nonanal and octane-4-methyl are also released by saline injected mice, thus these compounds are likely related to normal metabolic processes and amplified by the injection of DLD-1 cells. However, the increase of emission of these molecules depends on the injection method, being the ratio of abundance of nonanal respect to octane-4-methyl larger in subcutaneously injected mice. In addition, while the abundance of nonanal is not found dependent on both the injection method and the kind of injected cells, on the other hand, octane-4-methyl is produced by mice intravenously injected with scramble_RNAi_ cells.

In the intravenous experiment, GC-MS has been complemented by a gas sensor array that has been previously used for volatilome analysis in *in vivo* and *in vitro* experiments. In this study, sensor signals are in agreement with GC-MS data with the noteworthy difference that sensors begin capture the difference between scramble_RNAi_ and LOX-1_RNAi_ mice 2 weeks later with GC-MS.

At the current state of the art, the analysis of VOCs is mainly empirical, and the direct relationship between each VOC and tumor evolution mechanisms is still unclear. However, in this case the VOCs emission clearly correlates with the tumor evolution, providing a viable method for a simple, non-invasive alternative monitoring of tumor progression. Further studies are expected in this field to elucidate the intimate connection between VOCs and physiological processes.

Altogether, these results confirm the hypothesis that LOX-1 is a regulator of tumor progression, migration, invasion, metastasis formation, and tumor-related neo-angiogenesis, through the combination of specific molecular pathways. Further studies are necessary to deeper investigate the complex cancerogenesis mechanisms in which LOX-1 is actively involved.

## Data Availability Statement

All datasets generated for this study are included in the manuscript/Supplementary Files.

## Ethics Statement

This study was carried out in accordance with the ethical standards (Declaration of Helsinki), in compliance with Tor Vergata animal care guidelines and following national and international directives to minimize animal suffering (Italian Legislative Decree 26/2014, Directive 2010/62/EU of the European Parliament and of the Council). The protocol was approved by the Italian Ministry of Health (protocol no. 954/2016-PR).

## Author Contributions

MM, RCa, CP, RCi, and SP conducted the experiments and wrote the manuscript. AC and RP prepared the electronic nose. EM analyzed data. AO, RM, and GN wrote the manuscript. CD and FS analyzed data, conceived the work, and wrote the manuscript.

### Conflict of Interest

The authors declare that the research was conducted in the absence of any commercial or financial relationships that could be construed as a potential conflict of interest.

## Abbreviations

VOC: volatile compound
CRC: colorectal cancer
ox-LDL: oxidized-LDL
LOX-1: Lectin-like oxidized LDL receptor-1
VEGF: vascular endothelial growth factor
GC-MS: gas chromatography mass spectrometer
PCA: principal component analysis
H&E: Hematoxylin and Eosin
SPME: solid phase micro-extraction
QMB: quartz microbalances
SEM: standard error mean.
