# Supplementary material for: Targeting LOX-1 Inhibits Colorectal Cancer Metastasis in an Animal Model
Source: Front Oncol. 2019 Sep 19;9:927. doi: 10.3389/fonc.2019.00927 (PMC6761277; doi:10.3389/fonc.2019.00927)
Supplement: Supplementary file 1 [file Table_1.docx]

Supplementary Material

# Supplementary Data

Before using both LOX-1_RNAi_ and scramble_RNAi_ DLD-1 cells for mice injection, RT-qPCR analysis has been performed to further confirm the levels of LOX-1 mRNA expression (**Supplementary Figure 1**). As shown, DLD-1 cells, in which LOX-1 expression has been down regulated by shRNA, express lower level of LOX-1 transcript respect to those in which a scramble shRNA not targeting any human gene has been used (shRNAi scramble) (28). The difference resulted to be statistically significant.

DLD-1 cells have been injected subcutaneously (3.5x10^6^ of LOX-1_RNAi_ DLD-1 and 3.5x10^6^ of scramble_RNAi_ DLD-1) in the right flank of nude mice at 5-week-age (n=10 per group). In parallel a group of animals has been injected with a saline solution (n=10 per group). Mice body weight has been measured once a week, and no significantly differences have been observed (**Supplementary Figure 2*A***). No significant variance in the mean tumor volume has been revealed among two mice groups at day 6, 13, 20 and 28 from mass insurgence until mice sacrifice (**Supplementary Figure 2*C***), even if it has to be noted that tumor mass appeared in LOX-1_RNAi_ DLD-1 cells injected mice one week later respect to those mice injected with scramble_RNAi_ DLD-1 cells (**Supplementary Figure 2*C***). Moreover, after sacrifice, no differences among two mice groups have been observed in mean tumor weight, yet (**Supplementary Figure 2*B***)

## Supplementary Figures

**Supplementary Figure 1.** RT-qPCR for evaluating LOX-1 expression in scramble_RNAi_ and LOX-1_RNAi_ DLD-1. Results evidence a significant downregulation of LOX-1 mRNA in LOX-1_RNAi_ compared to scramble_RNAi_ DLD-1 cells. Data are representative of three independent experiments and reported as mean±SEM (**P*<0.05).

**Supplementary Figure 2.** Mice body weight **(A)**, mean tumor weight ***(B)*** and mean tumor volume **(C)** after subcutaneous injection of LOX-1_RNAi_ and scramble_RNAi_ DLD-1 cells. Any differences among LOX-1_RNAi_ and scramble_RNAi_ mice in terms of body weight, tumor weight and volume are statistically not significant. Data are represented as mean ± standard deviation; n=10 for each mice group. Saline refers to mice injected with saline solution.

**Supplementary Figure 3.** Statistical distribution (box-plot) of the abundances of most frequent VOCs released by subcutaneously injected mice for each group of mice (*Sal*-: mice injected with saline solution, *Scr*-: mice injected with scramble_RNAi_ DLD-1 cells_,_ *#5*: mice injected with LOX-1_RNAi_ DLD-1 cells). Numbers on *y*-axis correspond to different days from the injection (1, 6, 13, and 20) when VOCs have been collected. Abundance corresponding to each group of mice is described on *x*-axis and expressed as arbitrary unit. The feature in box-plots are the mean (red line), the standard deviations (the extremities of the box) and the outliers (crosses).

**Supplementary Figure 4.** Statistical distribution (box-plot) of the abundances of most frequent VOCs released by endovenously injected mice for each group of mice (*Sal*-: mice injected with saline solution, *Scr*-: mice injected with scramble_RNAi_ DLD-1 cells_,_ *#5*: mice injected with LOX-1_RNAi_ DLD-1 cells). Numbers on *y*-axis correspond to different weeks from the injection (0,4,6,9,11) when VOCs have been collected. Abundance corresponding to each group of mice is described on *x*-axis and expressed as arbitrary unit. The feature in box-plots are the mean (red line), the standard deviations (the extremities of the box) and the outliers (crosses).

**SUPPLEMENTARY TABLE 1.** VOCs found in cages of subcutaneously injected mice.

|  | retention time (minutes) | identified compound |
| --- | --- | --- |
| 1 | 3.61 | Isobutyl nitrite |
| 2 | 4.24 | Ethanol, 2-(1-methylethoxy)-' |
| 3 | 5.70 | Toluene |
| 4 | 6.73 | Hexanal |
| 5 | 7.02 | 4-Hepten-3-one. 5-methyl-' |
| 6 | 7.19 | Acetic acid, butyl ester |
| 7 | 7.41 | Heptane. 2.4-dimethyl- ' |
| 8 | 8.01 | Heptane. 3.4-dimethyl- ' |
| 9 | 8.72 | Octane, 4-methyl |
| 10 | 9.06 | Pyruvic acid, butyl ester |
| 11 | 9.46 | styrene |
| 12 | 9.60 | Unknown |
| 13 | 9.81 | Nonane |
| 14 | 10.32 | Acetyl valeryl |
| 15 | 10.53 | Propanoic acid, anhydride |
| 16 | 10.66 | 2,2,5-Trimethylhexan-4-one |
| 17 | 10.79 | alpha.-Pinene |
| 18 | 10.95 | 2,2,4-Trimethyl-3-pentanone |
| 19 | 11.14 | 2,3-Pentanedione ' |
| 20 | 11.36 | Pentane, 2,2-dimethyl-' |
| 21 | 11.65 | Decane |
| 22 | 11.80 | Hexane, 2,2,5,5-tetramethyl- ' |
| 23 | 11.99 | Octane, 4,4-dimethyl- |
| 24 | 12.37 | Heptanol' |
| 25 | 12.52 | unknown |
| 26 | 12.86 | Heptane. 2.5.5-trimethyl- ' |
| 27 | 12.99 | Octane, 2,4,6-trimethyl- |
| 28 | 13.20 | Nonane, 2,6-dimethyl- |
| 29 | 13.29 | Limonene |
| 30 | 13.48 | 'Butanoic acid, 1,1-dimethylethyl ester ' |
| 31 | 13.61 | unknown |
| 32 | 13.74 | Unknown |
| 33 | 13.77 | 7-Exo-ethyl-5-methyl-6,8-dioxabicyclo[3.2.1]oct-3-ene ' |
| 34 | 13.93 | Propanoic acid, 2-methyl, anhydride |
| 35 | 14.10 | Undecane |
| 36 | 14.23 | Octane, 5-ethyl-2-methyl- |
| 37 | 14.50 | Unknown |
| 38 | 14.72 | 1-Octanol, 3,7-dimethyl- |
| 39 | 14.90 | Unknown |
| 40 | 15.01 | Nonane, 5-butyl- |
| 41 | 15.17 | Nonanal |
| 42 | 15.30 | Hexane. 3.3-dimethyl- ' |
| 43 | 15.46 | Nonane, 5-(2-methylpropyl)- |
| 44 | 15.70 | Unknown |
| 45 | 15.75 | 3,4-Hexanedione, 2,2,5-trimethyl- ' |
| 46 | 15.93 | Decane, 3-methyl |
| 47 | 16.24 | Unknown |
| 48 | 16.57 | Undecane, 3,7-dimethyl- |
| 49 | 16.84 | Undecane, 2,9-dimethyl- |
| 50 | 17.36 | Dodecane |
| 51 | 17.49 | Decanal |
| 52 | 17.67 | Undecane, 3,6-dimethyl- |
| 53 | 17.84 | Dodecane, 4-methyl- |
| 54 | 18.18 | Undecane, 3,8-dimethyl- |
| 55 | 18.31 | Decane, 2,3,5-trimethyl- |
| 56 | 18.36 | Decane, 6-ethyl-2-methyl- |
| 57 | 18.54 | 3-Ethyl-3-methylheptane ' |
| 58 | 18.61 | Hexane. 2.4.4-trimethyl-' |
| 59 | 18.66 | Unknown |
| 60 | 18.73 | Decane, 2,3,8-trimethyl- |
| 61 | 18.98 | Unknown |
| 62 | 19.07 | Dodecane. 4.6-dimethyl-' |
| 63 | 19.24 | Dodecane, 2,7,10-trimethyl- |
| 64 | 19.35 | Decane, 2,3,6-trimethyl |
| 65 | 19.52 | Decane, 2,3,5,8-tetramethyl- |
| 66 | 19.62 | 2,4-Dimethyldodecane |
| 67 | 19.77 | Hexyl octyl ether |
| 68 | 20.18 | Unknown |
| 69 | 21.40 | Dodecane, 2,6,11-trimethyl- |
| 70 | 21.54 | Unknown |
| 71 | 21.84 | Tetradecane |
| 72 | 22.38 | Decane, 3-ethyl-3-methyl |
| 73 | 22.55 | Dodecane, 2,6,10-trimethyl- |
| 74 | 22.79 | 1,7-Dimethyl-4-(1-methylethyl)cyclodecane |
| 75 | 23.05 | Pentadecane |
| 76 | 23.20 | Decane, 2,3,5,8-tetramethyl- |
| 77 | 23.38 | Phenol. 3.5-bis(1.1-dimethylethyl)- ' |
| 78 | 24.94 | Hexadecane |
| 79 | 26.69 | heptadecane |

**SUPPLEMENTARY TABLE 2.** Recurrent compounds found in more than 80% of subcutaneously injected mice.


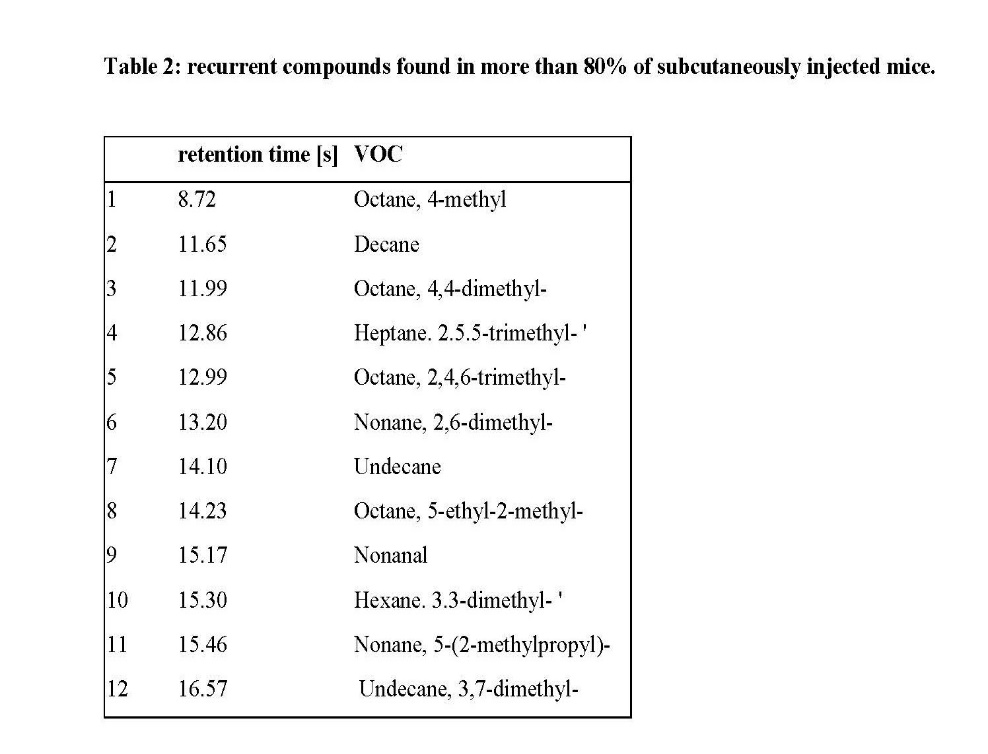


SUPPLEMENTARY TABLE 3. VOCs found in cages of endovenously injected mice

|  | retention time (minutes) | identified compound |
| --- | --- | --- |
| 1 | 3.61 | Isobutyl nitrite' |
| 2 | 4.24 | Ethanol, 2-(1-methylethoxy)-' |
| 3 | 5.70 | Toluene |
| 4 | 6.73 | Hexanal |
| 5 | 7.02 | 4-Hepten-3-one. 5-methyl-' |
| 6 | 7.19 | Acetic acid, butyl ester |
| 7 | 7.41 | Heptane. 2.4-dimethyl- ' |
| 8 | 8.01 | Heptane. 3.4-dimethyl- ' |
| 9 | 8.72 | Octane, 4-methyl |
| 10 | 9.06 | Pyruvic acid, butyl ester |
| 11 | 9.46 | styrene |
| 12 | 9.81 | Nonane |
| 13 | 10.32 | Acetyl valeryl |
| 14 | 10.53 | Propanoic acid, anhydride |
| 15 | 10.66 | 2,2,5-Trimethylhexan-4-one |
| 16 | 10.79 | alpha.-Pinene |
| 17 | 10.95 | 2,2,4-Trimethyl-3-pentanone |
| 18 | 11.14 | 2,3-Pentanedione ' |
| 19 | 11.36 | Pentane, 2,2-dimethyl-' |
| 20 | 11.65 | Decane |
| 21 | 11.80 | Hexane, 2,2,5,5-tetramethyl- ' |
| 22 | 11.99 | Octane, 4,4-dimethyl- |
| 23 | 12.37 | Heptanol' |
| 24 | 12.86 | Heptane. 2.5.5-trimethyl- ' |
| 25 | 12.99 | Octane, 2,4,6-trimethyl- |
| 26 | 13.20 | Nonane, 2,6-dimethyl- |
| 27 | 13.29 | Limonene |
| 28 | 13.48 | 'Butanoic acid, 1,1-dimethylethyl ester ' |
| 29 | 13.77 | 7-Exo-ethyl-5-methyl-6,8-dioxabicyclo[3.2.1]oct-3-ene ' |
| 30 | 13.93 | Propanoic acid, 2-methyl, anhydride |
| 31 | 14.10 | Undecane |
| 32 | 14.23 | Octane, 5-ethyl-2-methyl- |
| 33 | 14.72 | 1-Octanol, 3,7-dimethyl- |
| 34 | 15.01 | Nonane, 5-butyl- |
| 35 | 15.17 | Nonanal |
| 36 | 15.30 | Hexane. 3.3-dimethyl- ' |
| 37 | 15.46 | Nonane, 5-(2-methylpropyl)- |
| 38 | 15.75 | 3,4-Hexanedione, 2,2,5-trimethyl- ' |
| 39 | 15.93 | Decane, 3-methyl |
| 40 | 16.57 | Undecane, 3,7-dimethyl- |
| 41 | 16.84 | Undecane, 2,9-dimethyl- |
| 42 | 17.36 | Dodecane |
| 43 | 17.49 | Decanal |
| 44 | 17.67 | Undecane, 3,6-dimethyl- |
| 45 | 17.84 | Dodecane, 4-methyl- |
| 46 | 18.18 | Undecane, 3,8-dimethyl- |
| 47 | 18.31 | Decane, 2,3,5-trimethyl- |
| 48 | 18.36 | Decane, 6-ethyl-2-methyl- |
| 49 | 18.54 | 3-Ethyl-3-methylheptane ' |
| 50 | 18.61 | Hexane. 2.4.4-trimethyl-' |
| 51 | 18.73 | Decane, 2,3,8-trimethyl- |
| 52 | 19.07 | Dodecane. 4.6-dimethyl-' |
| 53 | 19.24 | Dodecane, 2,7,10-trimethyl- |
| 54 | 19.35 | Decane, 2,3,6-trimethyl |
| 55 | 19.52 | Decane, 2,3,5,8-tetramethyl- |
| 56 | 19.62 | 2,4-Dimethyldodecane |
| 57 | 19.77 | Hexyl octyl ether |
| 58 | 21.40 | Dodecane, 2,6,11-trimethyl- |
| 59 | 21.84 | Tetradecane |
| 60 | 22.38 | Decane, 3-ethyl-3-methyl |
| 61 | 22.55 | Dodecane, 2,6,10-trimethyl- |
| 62 | 22.79 | 1,7-Dimethyl-4-(1-methylethyl)cyclodecane |
| 63 | 23.05 | Pentadecane |
| 64 | 23.20 | Decane, 2,3,5,8-tetramethyl- |
| 65 | 23.38 | Phenol. 3.5-bis(1.1-dimethylethyl)- ' |
| 66 | 24.94 | Hexadecane |
| 67 | 26.69 | heptadecane |

**SUPPLEMENTARY TABLE 4.** Recurrent compounds found in more than 80% of subcutaneously injected mice.


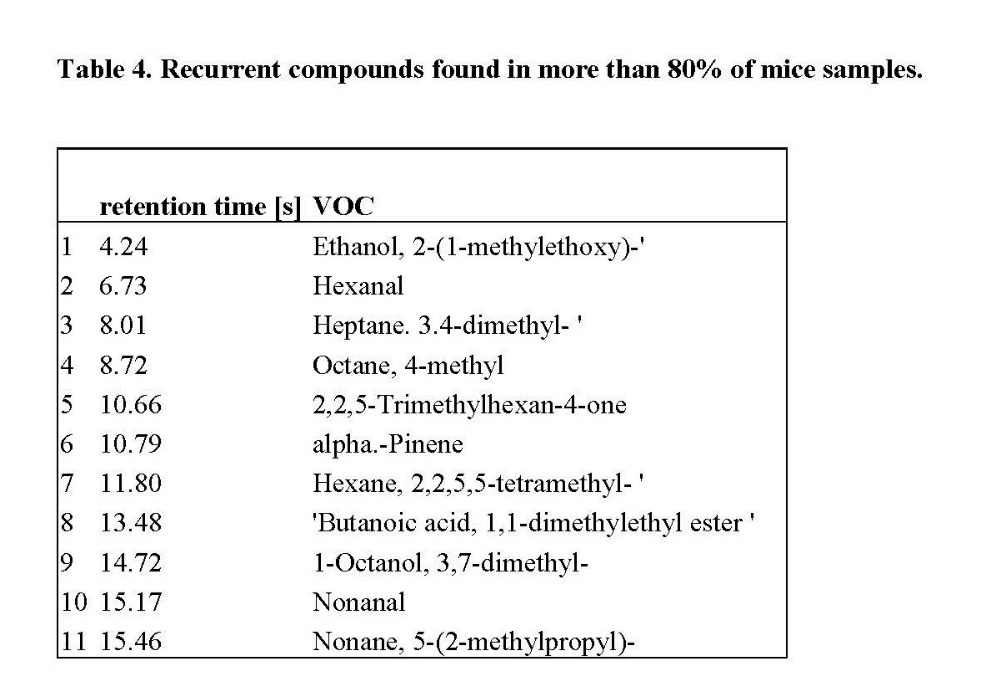


**Supplementary Methods**

*LOX-1 expression analysis*

Total RNAs from cells were extracted by Trizol Reagent (Invitrogen Life Technologies Corporation, Carlsbad, CA, USA) following manufacturer's instructions. Treatment with DNase I-RNase-free (Ambion, Life Technologies Corporation, Foster City, CA, USA) was used to eliminate genomic DNA contamination from total RNA samples. One μg of RNA was reverse transcribed with the High-Capacity cDNA Archive kit (Life Technologies Corporation, Foster City, CA, USA) and used in RT-qPCR. mRNAs were measured by SYBR Green (Life Technologies Corporation, Foster City, CA, USA) using specific primers, as reported in previous study (28).

*Subcutaneous Xenograft and Experimental Metastasis Mouse Models.*

Mice mean body weight has been monitored and xenograft growth volume has been calculated by measuring tumor volume in two dimensions by a caliper, following the formula: Tumor volume 4/3лr^3^; r = [length (mm) × width (mm)/4]. At the end point, specifically after 28 days from injection and when tumors reached approximately 1-2 cm^3^ in total volumes, tumor mass have been harvested and weighed.

*Gas Chromatography Mass Spectroscopy*

VOCs have been separated on the GC column using an initial oven temperature of 40 °C for 5 minutes, then increased by 7°C/min to 220°C, afterwards ramped by 15°C/min to 300°C that has been held for 3 min (total runtime: 39 min). Ultra-high purity helium has been used as carrier gas, working in linear velocity constant mode, with a carrier gas pressure of 24.9 kPa, total flow of 5.9 mL/min, column flow of 0.7 mL/min and linear velocity of 30.2 cm/s. The mass spectrometer is a single quadrupole analyser in electron ionization mode, set to record between 40 and 450 amu in the full scan mode. The temperature of transfer line and ion source is 250°C. The detector voltage has been set at 0.7 kV. GC-MS data have been analysed using the section GC-MS post-run analysis of the GC-MS solutions software (version 2.4, Shimadzu Corporation).
